# PKM2 Promotes Breast Cancer Progression by Regulating Epithelial Mesenchymal Transition

**DOI:** 10.1155/2020/8396023

**Published:** 2020-11-25

**Authors:** Hui Xiao, Longxiao Zhang, Yuan Chen, Chengjun Zhou, Xiao Wang, Dehai Wang, Zhenzhong Liu

**Affiliations:** ^1^The Second Hospital, Cheeloo College of Medicine, Shandong University, Jinan, Shandong 250033, China; ^2^The Affiliated Hospital of Qingdao University, Qingdao, Shandong 266003, China

## Abstract

Breast cancer is the leading cause of females characterized by high invasive potential. It is necessary to explore the underlying mechanism of breast cancer metastases and to find specific therapeutic targets. PKM2 is considered a new biomarker of cancer with upregulated expression in tumor tissue. PKM2 participates in the cancer-specific Warburg effect to regulate fast glucose intake consumption. Besides, PKM2 also contributes to cancer progression, especially tumor metastasis. In this study, we showed that PKM2 is upregulated in breast cancer tissues and the upregulating of PKM2 in breast cancer correlates with poor prognosis. PKM2 can regulate tumor progression by promoting tumor cell viability and mobility. Furthermore, overexpression of PKM2 can promote EMT to encourage tumor metastasis. These findings indicate PKM2 is a potentially useful diagnostic biomarker and therapeutic target in breast cancer.

## 1. Introduction

Breast cancer is the most frequently diagnosed cancer and the leading cause of cancer death among females [1]. It is stratified into three major subtypes: luminal, HER-2 positive, and TNBC (triple-negative breast cancer, estrogen receptor-negative, progesterone receptor-negative, and HER2-negative). Breast cancer, especially TNBC is characterized by high invasive potential. Approximately 30% of patients with early-stage breast cancer will develop metastases, with 5-year relative survival of 25% [2]. However, the molecular mechanisms underlying breast cancer development of different subtype are not clear, and the specific targetable biomarkers which can lead to the overall poor prognosis of the patients need to be further explored.

Activating invasion and metastasis is an important hallmark of cancer [3]. The metastasis of tumors is closely associated with epithelial-mesenchymal transition (EMT), which is an important step in carcinoma progression with the increasing ability to invade, to resist apoptosis, and to disseminate [4, 5]. In EMT, the typical epithelial histologic features are replaced by mesenchymal phenotypes including loss of cell-cell adhesion and cell polarity, downregulation of epithelial protein markers, and upregulation of mesenchymal markers [6]. Several signal pathways, including PI3K/AKT/mTOR, Wnt, and transforming growth factor *β* (TGF-*β*), participate in EMT. The complex interactions among cells, microenvironment, and multiple signaling pathways enable the transition from tumor in situ to aggressive and invasive carcinoma.

The roles of Pyruvate kinase M2 (PKM2) involving in cancer development have attracted considerable attention since Christofk et al. found that the PKM2 expression is necessary for cancer-specific aerobic glycolysis which is known as the Warburg effect [7]. Besides the tumor metabolic function, PKM2 also contributes to tumor metastasis, oncogenic cytokinesis, or tumor growth [8–11]. PKM2 also works as a protein kinase to regulate gene transcription by phosphorylating its substrates [12]. Previous studies have reported the significance of PKM2 in cancer cell growth and survival [11]. Therefore, understanding the biochemical functions of PKM2 in tumor progress will become crucial to find potential therapeutic targets and to develop novel therapies in both primary and metastatic TNBC.

In the present study, we found that PKM2 was upregulated in clinical breast cancer samples and correlated with poor prognosis. Our results suggested that the viability and mobility of breast cancer cells (MDA-MB-231 and MCF-7 cells) were regulated by PKM2. Moreover, we also showed that PKM2 mediates EMT to promote the migration and invasion of breast cancer cells by analyzing the EMT marker protein level and EMT transcription factor (EMT-TF) mRNA expression. Our findings have implications for the development of specific biomarkers and therapeutic drugs targeting PKM2.

## 2. Materials and Methods

### 2.1. Clinical Samples and Immunohistochemical Staining

This study was approved by the Ethics Committee of the Second Hospital of Shandong University, Jinan, China. The certificate number is KYLL-2018(LW)030. All of the patients or their guardians provided written consent. 66 breast cancer tissues (35 triple-negative, 15 luminal, and 16 HER-2-positive) and adjacent normal tissues derived from patients undergoing surgical procedures were fixed in ethanol and embedded in paraffin. Slides were incubated with anti-PKM2 primary antibody and then incubated with a secondary antibody. Antibody binding was visualized by incubating with the DAB kit (Solarbio, China). Immunohistochemical results of PKM2 in the tissue were evaluated independently by two pathologists blinded to the clinical data according to the semiquantitative immunoreactivity score (IRS). The IRS is calculated by the percentage of positive cells (4, >80%; 3, 51–80%; 2, 10–50%; 1, <10%; 0, 0%) and the intensity of the staining (3, strong; 2, moderate; 1, mild; and 0, no staining).

### 2.2. Cell Culture

The human breast cancer cell line MDA-MB-231 cells and MCF-7 cells were obtained from the American Type Culture Collection (ATCC). Cells were cultured in DMEM high glucose (Hyclone, USA) with 10% FBS (Gibco, USA) and 1% 100 U/mL penicillin-streptomycin (Gibco, USA) and maintained at 37°C in a humidified incubator containing 5% CO_2_.

### 2.3. RNA Interference

The small interfering RNA (siRNA) of PKM2 and negative control (NC) siRNA were synthesized in RiboBio Company (China). PKM2 and NC siRNA were transfected into MDA-MB-231 and MCF-7 cells by Lipofectamine™ RNAiMAX Transfection Reagent (Thermo, USA) according to the manufacturer's protocol.

### 2.4. EMT Induction

Cells were induced EMT as described before [8]. In brief, cells were seeded in a standard medium with 10% FBS for 48 h and then treated with 2.5 ng/mL TGF-*β*1 (Sigma-Aldrich). Next, they were incubated with 10 ng/mL EGF (Sigma-Aldrich, USA), 100× insulin-transferring selenium (ITS; Gibco, USA), and 50 nmol/L hydrocortisone (Sigma-Aldrich, USA) in FBS-free media for 48.

### 2.5. Cell Proliferation

The Cell Counting Kit (CCK-) 8 and colony formation assays were performed to evaluate the viability and proliferative capacity of MDA-MB-231 and MCF-7 cells in the absence or presence of siRNA to PKM2. In brief,2 × 10^3^ cells were seeded in 96-well plates and incubated for 48 hours, and then, CCK-8 (Beyotime, China) solutions were added to each well. After 30 minutes of incubation, the optical density of each well was measured at 450 nm using the Victor spectrophotometer (Thermo Fisher Scientific, USA).

### 2.6. Colony Formation Assay

1 × 10^3^ MDA-MB-231 and MCF-7 cells were seeded in a 6-well plate and cultured in complete medium in 5% CO_2_ at 37°C for 2 weeks. The cell colonies were stained by the crystal violet and then counted.

### 2.7. Cell Migration and Invasion

5 × 10^5^ cells in serum-free medium were seeded into the upper chamber of the transwell of 8 *μ*m pore size (Millipore, USA) while 10% FBS was added to the bottom chamber. After 12 h incubation, the cells were fixed with 4% paraformaldehyde and stained with 0.1% crystal violet. The cells on the upper surface of the filter were removed using a moistened cotton swab, and then, the cells of the lower surface of the filter were counted under a microscope (Nikon, Japan) at ×100 magnification. In invasion assay, the upper chamber was coated with 50 *μ*l of a matrigel solution before cell seeding.

### 2.8. Western Blot

Western blotting was performed in accordance with the standard protocols. In brief, cellular proteins were extracted using the RIPA lysis buffer, separated on SDS-PAGE gels, and transferred onto NC membranes (Millipore, Billerica, USA). After incubated in primary and second antibodies separately, proteins were detected by ECL reagent (Millipore, USA) and imaged with FluorChem Q (Protein simple, USA). The following antibodies were used for western blotting: PKM2 antibody (Proteintech, China, 1 : 1000, 15822-1-AP), vimentin antibody (Proteintech, China, 1 : 1000, 10366-1-AP), E-cadherin antibody (Proteintech, China, 1 : 1000, 20874-1-AP), N-cadherin antibody (Proteintech, China, 1 : 1000, 22018-1-AP), and GAPDH antibody (ZSGB-bio, China, 1 : 1000, TA-8).

### 2.9. Quantitative Real-Time PCR

The primers were synthesized by BGI (China), and the sequences are listed in Table 1. Total RNA was isolated from MDA-MB-231 using the RNA simple kit (Tiangen, China) following the manufacturer's instructions. cDNA was synthesized using the HiScript II Q RT SuperMix for qPCR (Vazyme, China), and quantitative real-time PCR (Q-RT PCR) was performed using Fastking One Step RT-PCR kit (Tiangen, China). GAPDH was used as the reference gene. The ∆∆Ct method was used to analyze the real-time PCR data.

### 2.10. Genomics Data Analyze

We used the analysis tool UALCAN to study the TCGA (http://cancergenome.nih.gov/) database of the expression level of PKM2 in breast cancer. Then, we used the Kaplan Meier plotter database (https://kmplot.com/analysis/) to analyze the correlations between PKM2 and prognosis.

### 2.11. Statistical Analysis

Three or more independent experiments were performed for each result, and the results were presented as mean ± S.D. Data analysis was performed with GraphPad Prism 5. Statistical analyses were performed using a two-tailed Student's *t*-test. *P* < 0.05 was considered to be significant.

## 3. Results

### 3.1. PKM2 Is Upregulated in Breast Cancer Tissues

**Figure 1:**
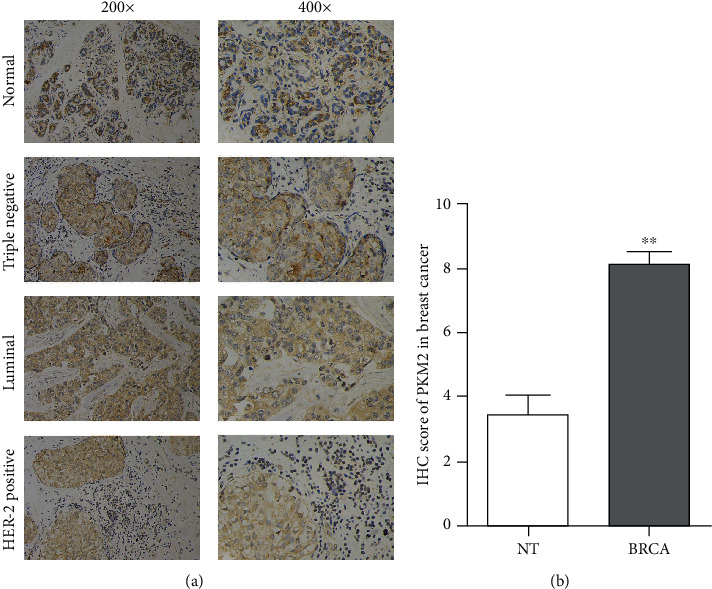
PKM2 is upregulated in breast cancer tissues. (a) Representative images of IHC staining of PKM2 in normal breast tissues and breast cancer tissue samples (TNBC, luminal, and HER-2 positive; ×200 and ×400 magnification). (b) IHC score of PKM2 in normal tissues (NT) and breast cancer (BRCA) (^∗∗^*P* < 0.01).

To investigate the expression level of PKM2 in breast cancer, we analyzed breast cancer samples by immunohistochemistry (IHC) and then assessed the staining. We found that PKM2 was significantly upregulated homogeneously in breast cancer tissues compared with normal adjacent noncancerous tissues, and the upregulation of PKM2 was in all 3 major subtypes (Figure 1(a) and 1(b)).

### 3.2. PKM2 Expression Associates with the Prognosis of Breast Cancer Patients

**Figure 2:**
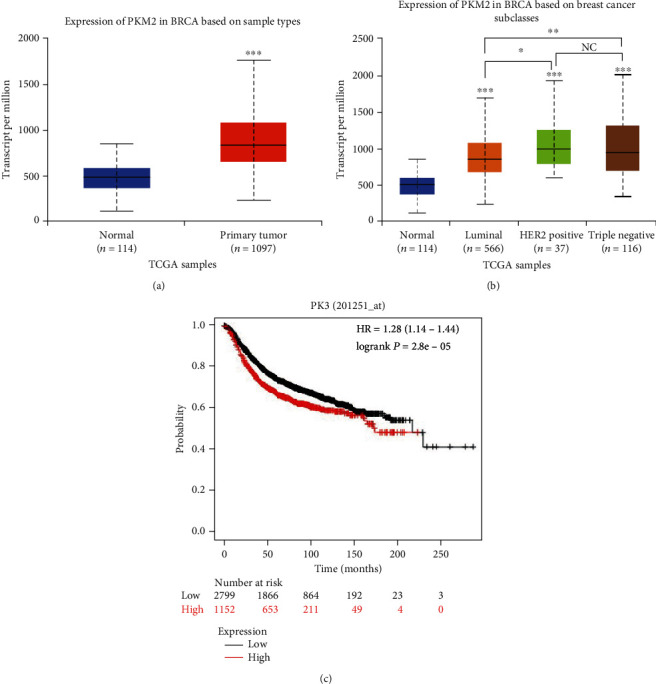
PKM2 expression associates with the prognosis of breast cancer patients. (a) Expression level of PKM2 in normal tissue and primary breast cancer tissues. (b) Expression level of PKM2 in different subclasses (TNBC, luminal, and HER-2) of breast cancer. (c) Kaplan–Meier curves showed that the patients with higher PKM2 expression had poorer overall survival (*P* < 0.005). Abbreviations: BRCA: breast invasive carcinoma (^∗∗∗^*P* < 0.001; ^∗∗^*P* < 0.01; ^∗^*P* < 0.05; NC : P > 0.01).

To further understand the clinical significance of PKM2 in breast cancer, we analyzed the PKM2 expression using UALCAN and patient prognosis using the Kaplan Meier plotter database. It revealed that PKM2 is upregulated in primary tumor tissues compared with adjacent noncancerous tissues (Figure 2(a)). PKM2 is overexpressed in different subclasses (luminal, HER-2 positive, and triple-negative) of breast cancer. Furthermore, we found that the expression of PKM2 shows no difference between HER2-positive and TNBC. However, there is a significant difference between luminal and HER-2 positive, or luminal and TNBC, according to the gene expression level analysis of the TCGA database (Figure 2(b)). The Kaplan–Meier survival analysis showed that breast cancer patients with high PKM2 expression had a poorer prognosis (Figure 2(c)). These results indicated that the upregulation of PKM2 in breast cancer tissues correlates with poor prognosis of patients, suggesting that PKM2 may play important roles during carcinoma progression.

### 3.3. PKM2 Is Required for Breast Cancer Cell Viability

**Figure 3:**
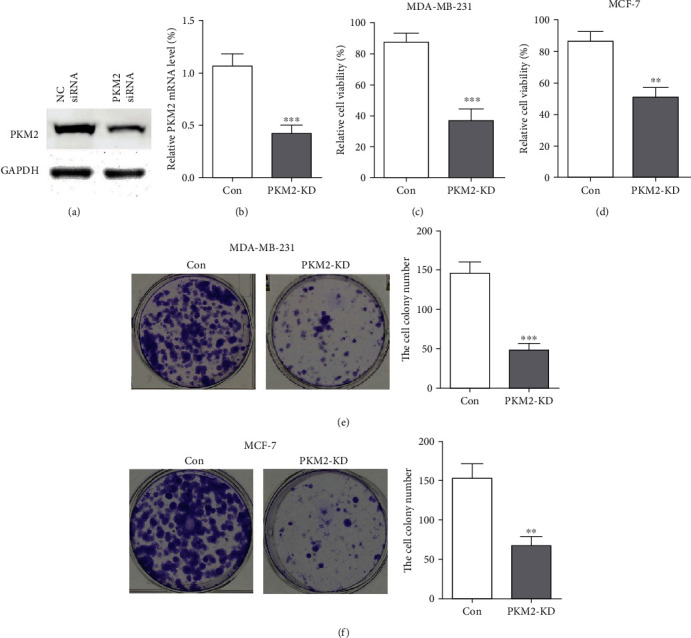
PKM2 is required for breast cancer cell viability. (a) PKM2 protein level was reduced by PKM2 siRNA, and GAPDH was used as a loading control. (b) Q-RT PCR analysis of PKM2 mRNA levels normalized to GAPDH in the control group and PKM2-KD cells. (c), (d) Cell viability was measured by CCK-8 assay in MDA-MB-231 and MCF-7 cells following PKM2 silencing by siRNA. (e), (f) Colony formation of PKM2-knockdown MDA-MB-231 and MCF-7cells was reduced (^∗∗∗^*P* < 0.001; ^∗∗^*P* < 0.01).

To determine the effect of PKM2 on cell viability, we knocked down the PKM2 expression by siRNA. The decreased protein levels of PKM2 in MDA-MB-231 cells were confirmed by western blot and Q-RT-PCR analysis compared with NC siRNA (Figure 3(a) and 3(b)). Then, CCK-8 and colony formation assays were performed to evaluate the viability and proliferative capacity of MDA-MB-231 and MCF-7 cells. Firstly, CCK-8 results showed that in the PKM2-knockdown (PKM2-KD) group, the cell viability was significantly decreased (Figures 3(c) and 3(d)). Colony formation assay is an in vitro cell survival assay based on the ability of a single cell to grow into a colony. The results showed that knockdown PKM2 significantly inhibited the number and size of all cell colonies of breast cancer cells compared to the control group (Figure 3(e) and 3(f)).

### 3.4. PKM2 Is Required for Breast Cancer Cell Migration and Invasion

**Figure 4:**
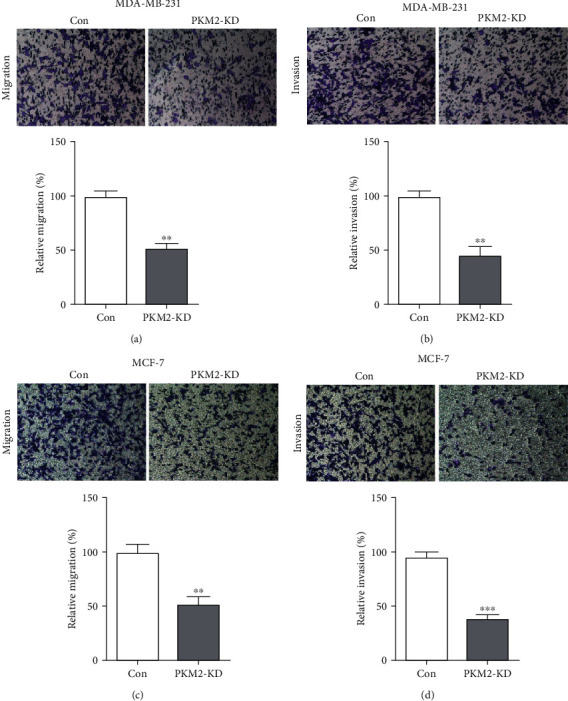
PKM2 is required for breast cancer cell migration and invasion. Cell migration (a) and invasion (b) of MDA-MB-231 cells after PKM2 knockdown was determined by transwell assay. Cell migration (c) and invasion (d) of MCF-7 cells after PKM2 knockdown was determined by transwell assay. Representative images from the migration and invasion of control and PKM2-KD cells of MDA-MB-231 and MCF-7 cells (top panel). Cell migration and invasion are expressed as a percentage of control (bottom panel). Cells were counted for at least five random microscope fields. Results are shown as mean ± SD from three independent experiments. (^∗∗∗^*P* < 0.001. ^∗∗^*P* < 0.01).

To evaluate the role of PKM2 on breast cancer mobility, the migrating and invasive ability of MDA-MB-231 and MCF-7 were measured after PKM2 knockdown using transwell. PKM2-KD cells migrated significantly slowly than the cells of the control group (Figure 4(a)). Furthermore, the invasive capacity of MDA-MB-231 cells was reduced in PKM2-KD cells (Figure 4(b)). The knockdown of PKM2 also inhibits the migration and invasion of the MCF-7 cells (Figures 4(c) and 4(d)). These results indicate that PKM2 has a vital role in promoting breast cancer cell migration and invasion.

### 3.5. Silencing PKM2 Suppresses Breast Cancer Cell EMT

**Figure 5:**
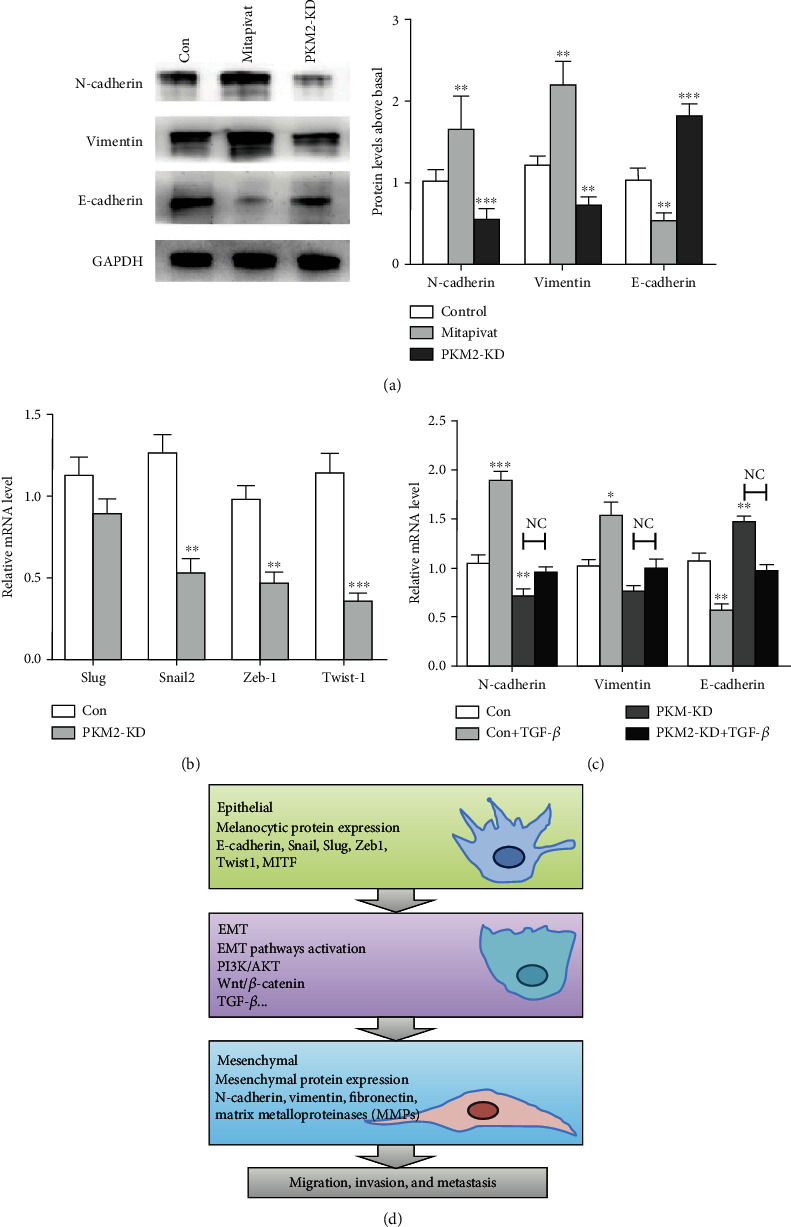
Silencing PKM2 suppresses MDA-MB-231 cell EMT. (a) Western blot analysis of protein levels of EMT marker proteins in control, Mitapivat, and PKM2-KD groups. GAPDH was used to normalize protein expression (left panel). The protein levels of EMT markers were quantified and compared (right panel). (b) Q-RT PCR analysis of mRNA levels of key EMT-related genes (Snail, Slug, Zeb1, and Twist1) in control and PKM2-KD cells normalized to GAPDH. (c) Relative mRNA levels of vimentin, N-cadherin, and E-cadherin after EMT induction in MDA-MB-231 cells in control and PKM2-KD cells for 48 h. (d) Changes involved in EMT (^∗∗∗^*P* < 0.001, ^∗∗^*P* < 0.01, NC : P > 0.01).

To determine how PKM2 controls the migratory and invasive phenotypes and whether PKM2 is responsible for the EMT of MDA-MB-231 cells, we evaluated the expression levels of the EMT biomarkers (vimentin, N-cadherin, and E-cadherin) by western blot after activating or knockdown of PKM2. These results showed that the activation of PKM2 by the specific activator, Mitapivat, resulted in increased mesenchymal markers (N-cadherin and vimentin) and decreased epithelial protein markers E-cadherin expression, while the knockdown of PKM2 blocked the E-cadherin loss and mesenchymal marker gain, which suggested that the EMT is inhibited (Figure 5(a)). Furthermore, we found that in MDA-MB-231 cells, PKM2 knockdown increased the expression of the key EMT-related genes (Snail, Slug, Zeb1, and Twist1) (Figure 5(b)). In a TGF-*β*1-induced EMT experiment, the results show that after the PKM2 knockdown, the MDA-MB-231 cells were less sensitive to TGF-*β*1, showing no significant alteration of the mRNA expression level of the key EMT markers to EMT induction (Figure 5(c)). This result suggested that PKM2 plays a vital role in EMT. Therefore, tumor cells gain mesenchymal morphology, and the promoted EMT increases cancer cell invasion and metastasis (Figure 5(d)). In brief, PKM2 promotes breast cancer cell EMT to regulate the migration and invasion in tumor progression.

## 4. Discussion

Our results showed that the TCGA database analysis indicated that the high PKM2 expression in breast cancer samples was correlated with reduced patient survival. PKM2 is upregulated in breast cancer tissues and proved that PKM2 can promote breast cancer progression by regulating tumor cell viability and motility. Furthermore, our results suggested that PKM2 is a key regulator of cell migration and invasion during breast cancer progression by promoting EMT.

Four isozymes of pyruvate kinase (M1, M2, R, and L) are differentially expressed in human tissue. PKM2 is expressed during embryonic development, while the PKM1 isoform is expressed in most adult tissues. However, reports showed that tumor tissues exclusively express PKM2 [13–15]. PKM2 locates both in the cytoplasm and nucleus, which illustrates its multiple functions in cancer cells. The allosteric switch between tetrameric form (high-activity state) and dimeric form (low-activity state) of PKM2 may lead to the variable functions of PKM2 as a pyruvate kinase and a protein kinase in cell metabolism [12, 15]. It was identified that PKM2 is regulated by several signals including EGF and hypoxia and then leading to downstream effects such as metabolic reprogramming and oncogene activation. PKM2 has an essential role in the Warburg effect, a cancer-specific glycolytic system that allows tumor cells to obtain energy rapidly to proliferate, migrate, and invade by converting glucose to lactate even when oxygen is abundant, meeting the biosynthetic demands for tumor development. Knocking down PKM2 and replacing it with PKM1 lead to reduced lactate production, increased oxygen consumption, and reduced tumor cell growth [7]. The allosteric switch between tetrameric form (high-activity state) and dimeric form (low-activity state) of PKM2 may lead to the variable functions of PKM2 in cell metabolism [15]. However, the nonmetabolic functions of PKM2 have remained controversial. Recent studies have revealed the new mechanisms of PKM2 in tumorigenesis, cell growth, survival, apoptosis, cancer stem-like properties, and EMT [8, 16–19]. Moreover, it has been proved as a useful diagnostic biomarker and therapeutic target in malignancies [20–22].

EMT is a biologic process during embryogenesis, organ development, and tissue regeneration [5]. Moreover, EMT is an important driver of cancer progression which has been shown to link closely to carcinoma metastasis [4]. Activating invasion and metastasis is an important hallmark of cancer [3]. A lot of literature showed that the metastasis of tumor is closely associated with EMT, which is an important step in the carcinoma progression with the increasing ability to invade, to resist apoptosis, and to disseminate [4, 5]. In EMT, the typical epithelial histologic features are replaced by mesenchymal phenotype including loss of cell-cell adhesion and cell polarity, downregulation of epithelial protein markers, and upregulation of mesenchymal markers [6]. Several signal pathways, including PI3K/AKT/mTOR, Wnt, and transforming growth factor *β* (TGF-*β*), participate in EMT. The complex interactions among cells, microenvironment, and multiple signaling pathways enable the transition from tumor in situ to aggressive and invasive carcinoma. Series molecular processes are engaged in EMT. As an early step, the loss of E-cadherin expression is the biochemical feature of EMT, inducing the activation of EMT-TFs including Snail, Slug, Twist1, and Zeb1. EMT-TFs which drive mesenchymal protein expression to permit subsequent invasion and metastasis by creating a protumorigenic environment [23, 24]. The mesenchymal protein markers, including N-cadherin, vimentin, fibronectin, and matrix metalloproteinases (MMPs), are upregulated, while the epithelial markers E-cadherin, desmoglein, and cytokeratin-18 are downregulated. Cancer cells that undergo EMT acquire epigenetic and genetic changes in expression of specific protein markers, morphology (from epithelial to fibroblastic-like and spindle-shaped), and function to enable invasion and metastasis (Figure 5(d)). Therefore, to find some small molecule inhibitors which specifically targeted EMT offers a novel approach to regulating tumor progression.

There is an increasing concern on the role of PKM2 in EMT. PKM2 expression and activity contribute to EMT in multiple cancers such as colon cancer, oral squamous cell carcinoma, esophageal squamous cell carcinoma, and so on [8, 25, 26]. The translocation from cytoplasm to the nucleus of PKM2 may promote EMT by regulating gene transcriptional activity. Therefore, we tested the contributions of PKM2 in EMT of breast cancer by analyzing the EMT marker protein expression. Our results indicated that PKM2 plays a vital role in tumor cell migration and invasion. After the knockdown of PKM2 by siRNA in MDA-MB-231 and MCF-7 cells, the ability to migrate and invade is inhibited. However, the underlying mechanism needs to be further explored.

Therefore, a specific drug targeting PKM2 is with great potential, for it can not only inhibit the Warburg effect to cut the energy source of the biosynthetic demands of cancer cells but also abolish the proliferation and metastasis of cancer cells to block the tumor progress.

As the most commonly diagnosed cancer and the leading cause of cancer death in women, breast cancer is recognized as a serious, worldwide health concern which is widely explored over the past decades, and great improvements have been made. Breast cancer, especially TNBC, exhibited a highly invasive behavior and enhanced the proliferating and migrating capacity of tumor cells. The application of multiple treatments partly decreases the overall mortality of breast cancer. Besides the systemic cytotoxic chemotherapy, targeted therapies make contributions in improving patient outcomes. However, owing to the highly aggressive behavior and metastatic rate of breast cancer, efforts to investigate the underlying mechanism and improve the outcome are still underway.

## 5. Conclusions

In our study, we focused on the bioactive role of PKM2 in EMT. The results proved that the upregulation of PKM2 in breast cancer samples is correlated with a poor prognosis of patients. The knockdown of PKM2 inhibits the proliferation, migration, and invasive capacities of breast cancer cells. Furthermore, our findings indicate that PKM2 contributes to EMT that activates the metastasis. The high-specific and low-toxic compounds targeting PKM2 are good candidates in cancer therapy. The results of our study have proved the role of PKM2 in EMT, which can have important clinical implications, providing a very promising biomarker in cancer diagnosis and targeted therapy.

## Figures and Tables

**Table 1:** Primer sequences for quantitative real-time PCR.

Gene name	Forward	Reverse
Zeb-1	GCTGGGAGGATGACACAGG	GTCCTCTTCAGGTGCCTCAG
Snail2	GAACTGGACACACATACAGTGAT	ACTCACTCGCCCCAAAGATG
Twist1	GCCGGAGACCTAGATGTCATT	TTTTAAAAGTGCGCCCCACG
Slug	CTGTGACAAGGAATATGTGAGCC	CAAATGCTCTGTTGCAGTGAG
N-cadherin	CCCTGCTTCAGGCGTCTGTA	TGCTTGCATAATGCGATTTCACC
Vimentin	CCACCAGGTCCGTGTCCTCGT	CGCTGCCCAGGCTGTAGGTG
E-cadherin	TTGCACCGGTCGACAAAGGAC	TGGAGTCCCAGGCGTAGACCAA
GAPDH	GCACCGTCAAGGCTGAGAAC	TGGTGAAGACGCCAGTGGA

## Data Availability

The data used to support the findings of this study are included within the article.

## Abbreviations

ATCC: American Type Culture Collection
BRCA: Breast invasive carcinoma
EMT: Epithelial-mesenchymal transition
EMT-TFs: EMT transcription factors
IHC: Immunohistochemistry
IRS: Immunoreactivity score
KD: Knockdown
NC: Negative control
PKM2: Pyruvate kinase M2
Q-RT PCR: Quantitative real-time PCR
siRNA: Small interfering RNA
TGF-*β*: Transforming growth factor *β*
TNBC: Triple-negative breast cancer.
